# Soft Tunable Lenses Based on Zipping Electroactive Polymer Actuators

**DOI:** 10.1002/advs.202003104

**Published:** 2020-12-23

**Authors:** Florian Hartmann, Lukas Penkner, Doris Danninger, Nikita Arnold, Martin Kaltenbrunner

**Affiliations:** ^1^ Division of Soft Matter Physics Institute for Experimental Physics Johannes Kepler University Altenberger Str. 69 Linz 4040 Austria; ^2^ Soft Materials Lab Linz Institute of Technology Johannes Kepler University Altenberger Str. 69 Linz 4040 Austria

**Keywords:** actuators, artificial muscles, electrostatic zipping, soft robotics, tunable lens

## Abstract

Compact and entirely soft optics with tunable and adaptive properties drive the development of life‐like soft robotic systems. Yet, existing approaches are either slow, require rigid components, or use high operating voltages of several kilovolts. Here, soft focus‐tunable lenses are introduced, which operate at practical voltages, cover a high range of adjustable focal lengths, and feature response times in the milliseconds range. The nature‐inspired design comprises a liquid‐filled elastomeric lens membrane, which is inflated by zipping electroactive polymers to tune the focal length. An analytic description of the tunable lens supports optimized designs and accurate prediction of the lens characteristics. Focal length changes between 22 and 550 mm (numerical aperture 0.14–0.005) within 260 ms, equal in performance to human eyes, are demonstrated for a lens with 3 mm aperture radius, while applying voltages below 500 V. The presented model, design rules, and fabrication methods address central challenges of soft electrostatic actuators and optical systems, and pave the way toward autonomous bio‐inspired robots and machines.

## Graphical abstract

Zipping electroactive polymer (ZEAP) actuators drive bioinspired tunable lenses with large focal length changes, are low‐cost, and fast. They achieve actuation speeds and focal length changes comparable to the human eye, switching the focus from 550 to 22 mm within 260 ms. Being overall soft, ZEAP tunable lenses advance bio‐mimetic vision systems and actuators.

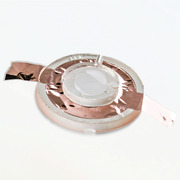

Conventional imaging optics for smartphones and cameras require systems of multiple lenses and motors to change focal length and provide variable magnification. However, the excessive number of components increases device complexity, weight, and price. Nature‐inspired optics that mimics the human eye enables soft tunable lens systems with reduced complexity, high compactness, and speed.^[^ ^1^, ^2^ ^]^ Realizing the essential functionality of an optical system with a single tunable lens represents a frugal approach with versatile potential applications, including vision systems for soft robotics, humanoids, or highly miniaturized optical systems.^[^ ^3^, ^4^ ^]^

Typically, tunable lenses take advantage of deformable lens‐shaped liquids, elastomers, or liquid‐filled elastic membranes, which change their focus with the help of electroactive transducers or materials. Commercially available lenses—based on electrowetting or reshaping rings—have liquid or soft optical components but require rigid encapsulations and actuators, which partially negate the advantages of soft materials.^[^ ^5^, ^6^ ^]^ Tunable lenses with micro‐pumps achieve high image quality with little aberrations, yet at the cost of size and compactness.^[^ ^7^, ^8^, ^9^ ^]^ Approaches utilizing inhomogeneous electric fields to reshape lens droplets potentially minimize the usage of rigid parts, but exhibit slow response times typically of the order of seconds.^[^ ^10^, ^11^ ^]^ Similarly, stimulus‐responsive soft materials such as hydrogels exhibit response times of tens of seconds as they are based on water diffusion or changing environments.^[^ ^12^ ^]^ Lenses tuned by dielectric elastomer actuators (DEAs) overcome all these limitations, as they operate at high speeds, are electrically driven and soft.^[^ ^1^, ^13^, ^14^, ^15^ ^]^ Such lens‐actuator systems closely mimic designs found in nature as the contraction of DEAs resembles ciliary muscles deforming the lens in the human eye.^[^ ^16^ ^]^ Their compactness and speed renders DEAs useful for such artificial (ciliary) muscles, with advantages over pneumatic actuators, thermally driven shape memory alloys or magnetically driven soft actuators, which usually require pumps, external heat sources or external magnetic fields.^[^ ^17^, ^18^ ^]^ Yet, DEAs require high driving voltages in the kilovolts range and suffer from dielectric breakdowns, which irreversibly damage the lens (Table S1, Supporting Information). Actuators based on the displacement of dielectric liquids via zipping electroactive polymers (ZEAP) survive dielectric breakdown and have promising applications as artificial muscles for soft robotics^[^ ^19^, ^20^ ^]^ and wearable haptic displays,^[^ ^21^ ^]^ but they require high driving voltages as well.

Here we introduce broadly applicable design rules, analytical models, and fabrication methods for soft tunable lenses based on ZEAP actuators operating at practical voltages. Zipping of thin metal‐coated plastic foils induces a hydraulic volume transfer, leading to shape change of elastic membranes at voltages below 500 V and focal length changes from 550 mm to as low as 22 mm, which equals a change of 0.005–0.14 in numerical aperture. The ZEAP actuators autonomously self‐heal from dielectric breakdown and allow fast focal length changes within milliseconds. Analytic and numerical models allow optimized designs and prediction of lens performances that agree well with the experiments. Our first results demonstrate powerful new types of soft tunable lenses for soft machines and robotics that are easily scalable, low‐cost, and allow for large focal length changes.

Our tunable lens architecture consists of a deformable elastomeric membrane in the center, circumvented by a circular ZEAP actuator (**Figure** **1**a). The membrane and the actuator share a reservoir of dielectric oil, which is radially injected toward the center when the polymer foils of the actuator zip, thus inflating the membrane. ZEAP actuators are built as metal‐insulator‐metal structures that use a dielectric liquid as insulator. In the off‐state, the dielectric liquid separates two metalized polymer foils—we here use 6‐µm thick polyethylene terephthalate (PET) metalized with 150 nm copper (Cu)—in balance with the lens membrane at its initial curvature (Figure 1b). Applying a voltage induces an electric field between the electrodes, which contract and start to zip, displacing the dielectric liquid. As the lens membrane has a low elastic modulus and thickness, it is inflated, resulting in a decreasing focal length and larger image magnification (Figure 1c). While the dielectric liquid is the main insulator in the off‐state, the polymer foil separates the metal electrodes when the actuator is zipped. Its thickness, permittivity, and initial opening angle mainly determine the operating voltage of the tunable lens. Here, we use an asymmetric electrode orientation to reduce the zipped capacitor thickness and operating voltage; the metallization of the PET‐foils faces downward for both electrodes (Cu‐PET‐Cu‐PET instead of the typically used Cu‐PET‐PET‐Cu). The size of the actuator determines the maximum amount of displaceable liquid and is readily adjusted without changing the fabrication methods (Figure 1d,e).

**Figure 1:**
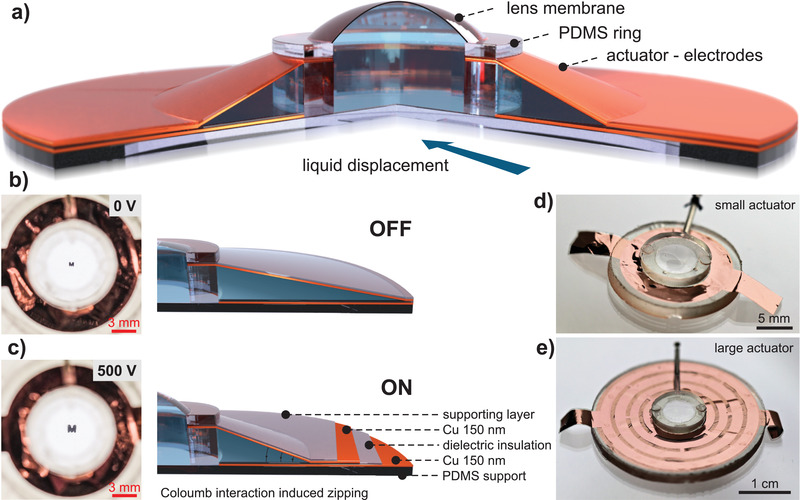
Tunable soft lenses with ZEAP actuators. a) Schematic of the tunable soft lens. Zipping of the actuator electrodes inflates the lens membrane in the center. b) In the off‐state the lens membrane is nearly flat and the dielectric liquid is evenly distributed. c) In the on‐state the metalized plastic foils contract due to the applied electric field, which inflates the lens membrane and magnifies the image (the letter “M”). d) Lens 3‐mm‐aperture radius and small actuator (16 mm diameter). e) Lens 3‐mm‐aperture radius and large actuator (30 mm diameter). The patterned electrodes aid concentric actuation.

As the lens performance depends on various design parameters such as membrane thickness and actuator dimensions, we modeled the lenses analytically and numerically to predict the focus versus voltage characteristics. The calculation is based on minimization of the system free energy *W*(Δ*V*), which separates into a load‐term, describing the elastic energy of the lens membrane, and an actuator‐term, representing the electrical energy in terms of voltage *U* and total capacitance *C* (details on the geometry are given in Figure S1 and Table S2, Supporting Information).

(1) $$\begin{array}{c} W=\int_{0}^{V_{0}+\Delta V}p\left(V\right)dV-C\left(\Delta V\right)\frac{U^{2}}{2} \end{array}$$

The first term here is elastic in nature, but it can be conveniently recalculated from the work–energy balance during the inflation, while the sign of the second term is negative under the voltage‐controlled conditions due to the work of the battery.^[^ ^22^, ^23^ ^]^ These two terms are coupled by the amount of displaced (incompressible) dielectric liquid Δ*V* = *V* − *V*_0_, which inflates the membrane starting from an initial state (off‐state) with volume *V*_0_ to volume *V* (voltage dependent on‐state) (**Figure** **2**a). Our analytic models approximate the membrane shape as a spherical cap, inflated by low amounts of displaced volume. This results in power laws for the pressure–volume (*p–V*) and focal‐length–volume (*f–V*) characteristics. For the numerical models, we use a system of ordinary differential equations with boundary conditions, based on a previous work,^[^ ^24^ ^]^ to describe the membrane shape and *p–V* characteristic. The paraxial focus is then obtained numerically from the membrane shape, applying Snell's law (for details, see Discussion in Supporting Information). Both analytic and numeric *p–V* characteristics result in power laws with exponent 3, with minor deviations at larger volumes (Figure 2b). An increasing aperture reduces energy required for a fixed volume change (given by the ∫*pdV*), and defines the working range of the tunable lenses.

**Figure 2:**
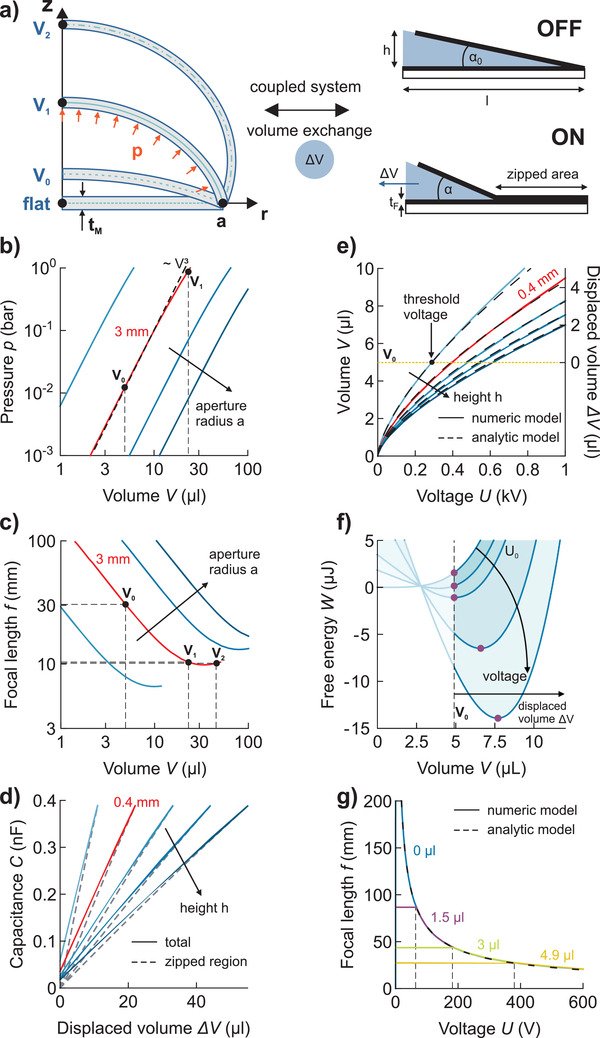
Modeling of soft tunable lenses. a) Applying a voltage to the actuator electrodes leads to a zipping induced volume exchange inflating the lens membrane (*t*_M_ = 0.2 mm, no pre‐stretch), which deforms from *V*_0_ = 4.9 µL to *V*_1_ = 23 µL and *V*_2_ = 45 µL. b) Pressure–volume characteristics follow a power law in the working range between *V*_0_ and *V*_1_. c) Focal‐length–volume characteristics for membranes with different aperture radii (2, 3, 4, and 5 mm, additional design parameters are fixed). Volumes larger than *V*_1_ have minor influence on the focal length. d) The capacitance of the electrodes is nearly linear in displaced volume, with dominant contribution from the zipped region. Characteristics are calculated for different actuator heights (0.2, 0.4, 0.6, 0.8, 1 mm, additional design parameters are fixed). e) The balance of mechanical and electrical forces yields the volume–voltage relations for different actuator heights. Initial fillings *V*_0_ lead to a threshold voltage that is required to displace volume from actuator to the lens membrane. f) The free energy of the system with boundary condition *V*_0_ exhibits a minimum at *V* > *V*_0_ when a voltage greater the threshold voltage (*U*_0_) is applied. g) Combining the results from (c) and (e) leads to the focal‐length–voltage characteristics for different initial volumes (0, 1.5, 3, 4.9 µL) and respective threshold voltages.

Inflation of the membrane decreases the focal length following a power law with exponent −1 in the working regime between *V*_0_ and *V*_1_ (Figure 2c). Beyond this range, further displaced volume results in overall membrane stretch only, with little curvature changes around the apex.

The actuator, in electrical terms a simple capacitor, delivers the energy required to inflate the membrane. Its capacitance *C*, with contributions from the zipped and unzipped region, almost linearly changes with displaced volume (Figure 2d). Previous calculations for Peano‐HASEL actuators (a special form of uniaxial ZEAP actuators) assume that the capacitance is mostly determined by the zipped region alone.^[^ ^25^ ^]^ While this assumption gives a good approximation for large actuator heights *h* (hence large opening angles of the electrodes), the contribution of the unzipped region increases for small heights and cannot be neglected near the fully unzipped state. In a general description, the minimization of free energy (1) with respect to displaced volume Δ*V* (internal variable of the system) results in force balance for any ZEAP tunable lens, which can be written as:

(2) $$\begin{array}{c} p\left(V_{0}+\Delta V\right)=\frac{dC}{d\Delta V}\frac{U^{2}}{2} \end{array}$$

Here, *U* is the applied voltage. This equation relates the applied voltage to the displaced volume with good agreement for both numerical and analytic approaches (Figure 2e). The final focal‐length–voltage characteristic *f*(*U*) is then obtained recalculating the volume *V* into focal length *f* for a given geometry, resulting in:

(3) $$\begin{array}{c} f\left(U\right)=\frac{\pi a}{4\left(n-1\right)}{\left(\frac{U_{c}}{U}\right)}^{2/3} \end{array}$$

Here, *U*_c_ is the characteristic voltage, which includes the majority of material and design parameters except basic scaling with aperture size *a*, refractive index *n*, and *V*_0_, and is a quantity that relates to the operating voltage (see Discussion in Supporting Information). An essential feature of the system is the emergence of a critical (threshold) voltage *U*_0_ = (*V*_0_/*a* ^3^)^3/2^ *U*_c_, below which no changes take place. The free energy has a minimum at *V* > *V*_0_ only if *U* surpasses this threshold voltage (Figure 2f). Both the initial focal length *f*_0_ and threshold voltage *U*_0_ are determined by the initial filling volume *V*_0_, which limits *f* from above. The threshold vanishes for *V*_0_ = 0 (no filling volume), where *f*_0_ becomes infinite. For lenses with 3‐mm aperture radius and a 200‐µm‐thick membrane, the focal length is readily tuned with voltages below 500 V and threshold voltages are below 200 V for initial fillings up to 3 µL (Figure 2g).

Design of sub‐kV tunable lenses with high range of focal length changes is based on guidelines from our models, while choosing inexpensive commodity materials. Our theoretical analysis provides design routes that yield even lower operating voltages (<100 V) and are outlined in the Supporting Information. The fabrication starts with thermal evaporation of 150 nm Cu on 6 µm thick PET‐foil. Where no electrodes are needed, Cu is removed with laser‐induced ablation, and the PET‐foil is subsequently cut in shape by laser machining. A bottom elastomer base for structural support and the lens membrane are mold‐cast from polydimethylsiloxane (PDMS) using 3D printed molds with polymethyl methacrylate (PMMA) covers (**Figure** **3**a,b). This results in an overall soft structure that is compressible and flexible (Figure S2, Supporting Information). Double‐sided 50‐µm‐thick elastic adhesion tape connects the individual parts of the lens, which are readily stacked on top of each other (Figure 3c). The connection of lens membrane and base keeps the membrane in a fixed position and allows homogenous inflation (and pressure) by the dielectric fluid. Omitting this connection can lead to directional inflation of the membrane, which is beneficial for other applications that involve touch, such as buttons or brail displays.^[^ ^21^ ^]^ Heat‐sealing the electrodes in regular steps along the edge of the electrodes introduces specific starting points for zipping to improve the zipping homogeneity (we use this for all our lenses) and partially reduces the threshold voltage (Figure S3, Supporting Information). The dielectric fluid is a low viscosity paraffin oil with a refractive index *n* = 1.48 (close to PDMS); it is injected with a syringe through an access tube and sealed. Lenses with a larger actuator are produced without changing the fabrication methods but require—in addition to heat‐seals— structuring of the electrodes to achieve more homogeneous zipping (Figure S4, Supporting Information). We realize this by engraving concentric rings into the electrodes, which results in ring‐by‐ring zipping and nearly concentric zipping fronts (Figure S5, Supporting Information). The total material costs for a single lens are about 14 cents and are majorly (96%) dominated by the PDMS elastomer (see Discussion and Table S3 in Supporting Information).

**Figure 3:**
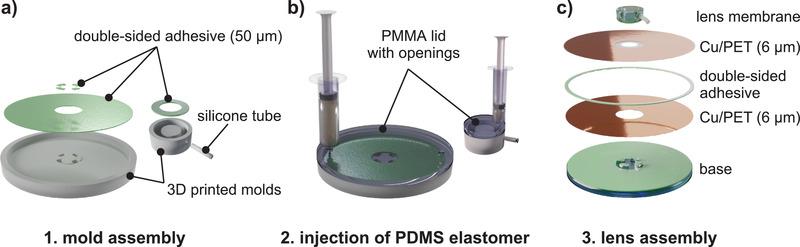
Soft lens fabrication and assembly. a) 3D printed molds for the lens membrane and base. Double‐sided adhesive is loosely applied within the molds to allow easy lens assembly. b) Molds are covered with PMMA lids, and PDMS elastomer is injected through one of the access holes. c) The lens is assembled together with the actuator electrodes using adhesive tape and filled with paraffin oil through the attached silicon tube.

For a 3‐mm aperture radius lens, an actuator with 16 mm diameter is sufficient to inflate the lens membrane to full extent (maximum focal length change). Depending on the filling level, the initial focal length is freely adjusted; here we present three lenses with ≈50, 110, and 550 mm initial focal length (**Figure** **4**a–c and Figure S6, Supporting Information). According to the models, the focal length remains constant below the threshold voltage, which decreases for smaller initial fillings (larger maximal focal length). However, the experimental behavior below and near the threshold is influenced by locally varying zipping, caused by the heat seals or other inhomogeneities (Figure 4a–c). A voltage of 450 V reduces the focal length to about 22 mm for all lenses, in agreement with our models. Deviations from the model occur in the mid‐voltage range (100–200 V) for the lens with the largest initial focal length (Figure 4c). We hypothesize that lower initial fillings lead to less uniform opening angles of the electrodes and less concentric zipping. This effect is also visible for the lenses with large actuators, which feature smaller electrode opening angles and therefore potentially lower operating voltages (Figure S7, Supporting Information), albeit with a less ideal zipping behavior.

**Figure 4:**
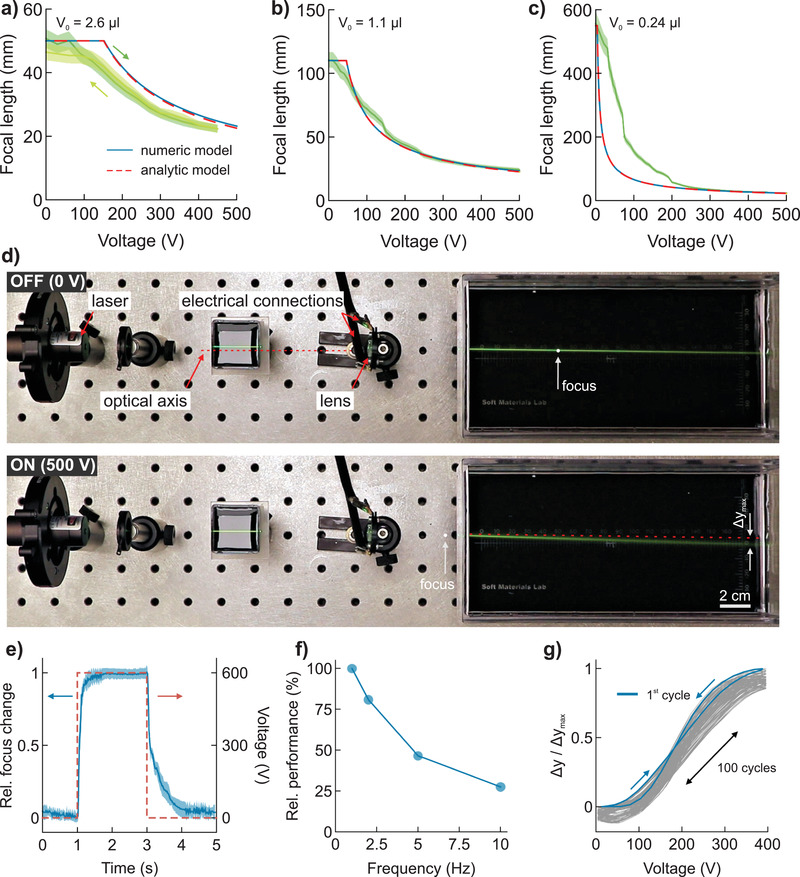
Lens performance. a–c) Measured focal‐length–voltage characteristics in comparison to numerical (blue) and analytic models (red dashed) for lenses with different initial focal lengths. d) Laser beam deflection through the tunable lens during operation. The maximum deflection is denoted as Δ*y*_max_. e) Response to step signal with 600 V amplitude is averaged over 100 cycles and results in 260 ms to reach 95% rel. focus change. Error band, standard deviation for 100 cycles. f) Relative performance as a function of frequency for triangular wave driving voltages with 400 V amplitude. g) Relative deflection change for 100 continuous actuation cycles.

The dynamic performance of the lenses is tested by deflecting a laser beam passing slightly off the optical axis (Figure 4d, Figure S8, Supporting Information). The relative change of the tangent of the beam angle with the optical axis, or the relative laser spot position at any image plane, is proportional to the relative focal length change (see Discussion in Supporting Information). Applying a step function with 2 s pulse width and 600 V amplitude visualizes the rates for switching lenses from the on‐ to the off‐state, averaged over 100 switching cycles (Figure 4e). The switching to the on‐state is faster; it requires about 260 ms to reach about 95% of the maximal focal length change. Once switched on, the lens retains its focus for the duration of the pulse. When switched off, it takes about 500 ms to return to within 15% change from the initial focal length change. This performance is comparable to a young human eye (20–30 y), which achieves 240 ms reaction time for near accommodation.^[^ ^26^ ^]^ Switching to the on state is inherently faster, since this process is electrically driven by the actuator, while switching to the off state is a passive process governed by the mechanic relaxation of the system. The frequency‐dependent performance of the lenses is tested by applying triangular waves with 400 V amplitude at different frequencies. We quantify the performance by the fraction of the beam deflection to its maximum possible value Δ*y*/Δ*y*_max_, and define the performance at 1 Hz as 100%. Increasing the frequency decreases the lenses performance, which however remains above 25% at 10 Hz (Figure 4f). The relative deflection of the laser beam shows little hysteresis during 100 repeated actuation cycles actuation with 1 Hz, yet a slight drift is visible, which however stabilizes after a few 100 cycles (Figure 4g, Figure S9, Supporting Information). Enhanced lens control can be achieved using the capacitance—capacitance and displaced volume relate nearly linearly—as additional control parameter in a closed‐loop^[^ ^27^ ^]^ and provides a possible solution to compensate drifts.

We report a versatile strategy to fabricate entirely soft electrically tunable lenses that operate at voltages below 500 V. Analytic and numerical models that describe the combined deformable lens‐actuator system are presented, which can be applied to a wide range of ZEAP‐actuated systems. The focal length can be rapidly changed between 550 and 22 mm within milliseconds. With the current design and materials, the lens maintains 25% of its performance even at switching speeds as high as 10 Hz. Even faster actuation speeds are possible by choosing dielectric liquids with lower viscosity, and optimizing the fluidic channels within the lens. Usage of plastic foils with either lower thickness or higher permittivity further reduces the operating voltages. Our approach represents a major step towards low‐voltage electroactive actuators, applicable to tunable optics, soft machines and robotics.

## Experimental Section

##### Materials

All chemicals were used as received without further purification. Polydimethylsiloxane, PDMS (Sylgard 184, Dow‐Corning, USA) was used for the lens body and membrane. Dow‐Corning Primer (DOWSIL 1200 OS Primer, Dow‐Corning, USA) and acrylic elastomer adhesive tape (VHB 467MP, 3M, USA) were used to bond PDMS to the PET foils of the actuator. Paraffin oil (Paraffin oil thin, Roth, Germany) was used as a liquid dielectric.

##### Base and Membrane Fabrication

High precision molds were 3D printed with a stereolithography printer (Form 3, Formlabs, USA) and post‐cured at 60 °C under UV light. VHB adhesive tapes were laser cut (Speedy 300 flexx, Trotec, Austria), coated with a thin layer of primer using an airbrush gun, and fixed within the molds. The molds were closed with PMMA covers, which feature two access holes for polymer injection. The membrane molds additionally feature a lateral hole, where a small silicon tube was mounted and sealed. PDMS prepolymer and cross‐linker were mixed 10:1 in a planetary mixer (SpeedMixer DAC400, Hausschild, Germany) under vacuum, injected into the molds with a syringe, and cured at 65 °C for at least 6 h.

##### Electrodes Fabrication

150 nm Cu was thermally evaporated (Univex350, Leybold, Germany) on 6‐µm thin PET foil, with 3 nm of chromium as adhesion layer. The electrodes were structured using laser ablation and laser cut into the desired shapes.

##### Lens Assembly

Base, electrodes, and lens membrane were stacked together utilizing the cast‐in adhesives, with an additional ring of VHB between the two electrodes. Spot welding was used for all lenses to introduce starting points for zipping fronts. Therefore, the electrodes were covered with 75‐µm‐thick polyimide and the 185 °C‐hot nozzle of a 3D printer (Ultimaker 3, Ultimaker, Netherlands) was used for welding (10 s). All lenses were filled and vented with paraffin oil via the silicon tube and sealed in advance.

##### Focal Length Measurement

The lens was placed under an objective (8×) of a microscope (Micromanipulator, model 6200), equipped with a digital camera (Bresser, MikroCam SP 5.0). A micrometer scale served as image to measure the magnification, from which the focal length was calculated. A function generator (33250A, Agilent Technologies, USA) and a voltage amplifier (PD05034, Trek Inc., USA) drove the lens, applying a 50 Hz bipolar square wave signal with voltages up to ±450 V.

##### Response Characterization

A laser beam (wavelength 450 nm) with slight offset to the optical axis was directed through the lens and basins filled with water‐diluted rhodamine 6 g. The lens was driven with a voltage amplifier, which was controlled by a function generator. A step function of 2 s pulse width (5 s period) and 600 V amplitude was applied to investigate the response behavior. For dynamic characterization a 400 V (1–10 Hz) triangular wave signal was applied. The experiments were recorded at 50 fps with a camera and evaluated via beam tracing.

## Conflict of Interest

The authors declare no conflict of interest.

## Author Contribution

F.H. and M.K. conceived the research project; N.A., F.H., and L.P. derived analytic and numerical models; L.P. and F.H. designed and fabricated the tunable lenses; F.H. and L.P. designed the experiments; L.P. conducted the characterizations; F.H., L.P., and D.D. prepared the figures and tables; F.H., L.P., D.D., and N.A. wrote the manuscript; all authors contributed to editing the manuscript; F.H. and M.K. supervised the research.

## Supporting information

Supporting Information

Supplemental Video 1

Supplemental Video 2

Supplemental Video 3
