# Supplementary material for: Soft Tunable Lenses Based on Zipping Electroactive Polymer Actuators
Source: Adv Sci (Weinh). 2020 Dec 23;8(3):2003104. doi: 10.1002/advs.202003104 (PMC7856880; doi:10.1002/advs.202003104)
Supplement: Supplementary file 1 — Supporting Information [file ADVS-8-2003104-s001.pdf]

© 2020 Wiley-VCH GmbH

ADVANCED SCIENCE
Open Access

Supporting Information

for *Adv. Sci.,* DOI: 10.1002/advs.202003104

Soft tunable lenses based on zipping electroactive polymer actuators

Florian Hartmann, Lukas Penkner, Doris Danninger, Nikita Arnold, and Martin Kaltenbrunner

Supplementary Information

Soft tunable lenses based on zipping electroactive polymer actuators.

Florian Hartmann[1,2], Lukas Penkner[1,2], Doris Danninger[1,2], Nikita Arnold[1,2], and Martin Kaltenbrunner[1,2]

[1]Division of Soft Matter Physics, Institute for Experimental Physics, Johannes Kepler University Linz, Altenberger Strasse 69, 4040 Linz, Austria

[2]Soft Materials Lab, Linz Institute of Technology, Johannes Kepler University Linz, Altenberger Strasse 69, 4040 Linz, Austria

Correspondence to: martin.kaltenbrunner@jku.at

This PDF file includes:

Supplementary Discussion

Supplementary Tables 1-3

Supplementary Figures 1-9

Supplementary Discussion

Total free energy of a tunable soft lens with actuator

The (free) energy W of the whole tunable lens system splits into the energy of the lens membrane W_{M} and the energy of the actuator W_{A}.

$$W = W_{\mathrm{M}} + W_{\mathrm{A}} \tag{1}$$

In a quasi-static description, which we assume to suffice for moderate operating speeds, we neglect dynamic effects, such as kinetic energy and the acceleration of the dielectric liquid and the ambient medium (air in our case). Strategies for analysis of dynamic effects can be found in the literature [1,2]. The elastic energy of the membrane resides in the stretched elastomer, but it can be calculated from the integral of its pressure-volume (p-V) characteristic, where the initial filling level V_0 defines the reference state. In this reference state, no voltage is applied, and the actuator/lens membrane system is in balance, due to equilibrium between the over-pressure and elasticity. In the actuated state, the electrodes of the actuator are connected to a constant voltage source, which then zip, and inflate the lens membrane to filling levels $V > V_0$. The difference in volume $\Delta V = V - V_0$ is called displaced volume, it is the volume exchanged between the actuator and lens membrane. We consider only reference states with convex lens shapes, hence $V_0 \geq 0$, however states with $V_0 < 0$ can be experimentally realized and modelled within our framework. We calculate the elastic (free) energy of the incompressible lens membrane as

$$W_{\mathrm{M}} = \int_0^{V_0+\Delta V} p(V)\,\mathrm{d}V \tag{2}$$

To derive the actuator energy W_{A}, we model the actuator as a radial-symmetric capacitor, surrounding the lens membrane. Its capacitance $C(\Delta V)$ changes, as the actuator zips. W_{A} is dominated by the electrostatic contributions; the elastic energy of the actuator is much smaller than for the lens membrane, because the product of its material stiffness and thickness is one order of magnitude larger. The electrostatic energy of the capacitor $W_{\mathrm{A'}}$ is:

$$W_{\mathrm{A'}} = \frac{Q^2}{2C} \tag{3}$$

As the actuator zips, its capacitance increases, and the charges are provided by the power supply with the constant voltage U. The work of the external power supply QU should be subtracted in the energy balance, which reverses the sign of the electrostatic contribution to *free* energy [3, 4], which then becomes:

$$W_{\mathrm{A}} = -\frac{CU^2}{2} \tag{4}$$

The total free energy of the system (up to an arbitrary constant) is then written as[1]

$$W = \int_0^{V_0+\Delta V} p(V)\,\mathrm{d}V - \frac{U^2}{2} C(\Delta V) \tag{5}$$

The equilibrium condition is provided by the minimum of this expression with respect to the internal system variable ΔV, resulting in

$$p(V_0 + \Delta V) = \frac{U^2}{2} \frac{d\,C(\Delta V)}{d\Delta V} \tag{6}$$

In the next sections, we derive expressions for $p(V)$ and $C(\Delta V)$ and provide an analytical model for the focus-voltage characteristics of ZEAP tunable lenses.

[1] The *elastic* energy of the dielectric fluid can be neglected, as it is considered to be incompressible. Its compression response is much stiffer, than the elastic response of the membrane, which defines the lens volume. Under such conditions, all the elastic energy is stored in the softer material.

Inflation of a planar elastomer-membrane with prestretch

Consider a thin circular elastic membrane with initial radius a and *relaxed, unstretched* thickness t_{M}, which is clamped along its circumference, and is inflated by a uniform pressure p. The z-axis is the symmetry axis and r is the polar radius. In general case, the membrane can have an initial lateral equal-biaxial prestretch λ_0. The height of the inflated membrane in z-direction is denoted as Z[2], its area as S, and the enclosed volume as V.
Assuming that the inflated membrane is a spherical cap, these quantities can be written as

$$S = \pi \left(a^2 + Z^2\right) = \pi \frac{a^2 \lambda^2}{\lambda_0^2} \tag{7}$$

$$V = \frac{\pi h}{6}\left(3a^2 + Z^2\right) = \frac{\pi a^3}{6}\left(2 + \frac{\lambda^2}{\lambda_0^2}\right)\sqrt{\frac{\lambda^2}{\lambda_0^2} - 1} \tag{8}$$

Here, λ is the average stretch, based on the area change with respect to its initial value (which may includes a prestretch λ_0). From eq. (7) we obtain:

$$\lambda = \lambda_0 \sqrt{1 + Z^2/a^2} \tag{9}$$

Using the work-energy balance $p\mathrm{d}V = V_{\mathrm{M}}\mathrm{d}G$, where V_{M} is the (constant) volume of the (thin, incompressible) membrane, we can relate the (over)pressure to the derivative of the elastic energy density G_λ

$$p = V_{\mathrm{M}} \frac{\mathrm{d}G}{\mathrm{d}V} = \frac{\pi a^2 t_{\mathrm{M}}}{\lambda_0^2} \frac{G_\lambda}{\mathrm{d}V/\mathrm{d}\lambda} = \frac{2\lambda_0 \sqrt{\lambda^2 - \lambda_0^2}}{\lambda^3} \frac{t_{\mathrm{M}}}{a} G_\lambda \tag{10}$$

The expression (10) is rather general.[3] The $p(V)$ dependence can be simplified for small stretches without prestretch, where the Neo-Hookean relation holds for G and $\lambda = 1 + \delta\lambda$, $\delta\lambda << 1$. This case is the most relevant for elastomer lens modelling. With the shear modulus μ, the derivative of elastic energy density is written as

$$G_\lambda = 2\mu\left(\lambda - \lambda^{-5}\right) \approx 12\mu\, \delta\lambda \tag{11}$$

This results in simplified expressions for the pressure, while the volume can be obtained from the eq. (8)

$$p \approx 24\sqrt{2}\, \frac{\mu\, t_{\mathrm{M}}}{a} \delta\lambda^{3/2} \tag{12}$$

$$V \approx \pi a^3 \sqrt{\frac{\delta\lambda}{2}} \tag{13}$$

Combining both equations results in the pressure-volume characteristics for the initial stage.

$$p(V) = \frac{3 \times 2^5}{\pi^3} \frac{\mu\, t_{\mathrm{M}}}{a} \left(\frac{V}{a^3}\right)^3 \tag{14}$$

This approximations assumes small homogeneous stretch.[4] In reality, the stretch and membrane thickness vary along the membrane, and its shape deviates from a spherical cap. A more accurate

[2]It is often convenient to use Z as an underlying parametrizing variable instead of V or λ, especially in the intermediate calculations.

[3]It is instructive to compare it with the similar result for a spherical balloon with initial unstretched radius a, $p = t_{\mathrm{M}} G_\lambda / a\lambda^2$.

[4]With prestretch, the dependence (14) changes drastically, and becomes linear in volume V. This may be used for better control of the system, but requires more rigid design parts, which we aimed to avoid in this work.

fast calculation for the p-V-characteristic can still be performed numerically within the frame of linear material elasticity.[5]

Numeric calculations for the inflation of a circular elastic membrane are based on a model from Pokorny *et al.* [5]. The applied pressure deforms the membrane such, that a point with initial coordinates $(r, 0)$ moves to the position $(r + u(r), w(r))$ (Supplementary Fig. S2). Thus, $u(r)$ and $w(r)$ represent the radial displacement and the deflection in z-direction. An initial prestretch λ_0 of the membrane causes a radial stretch λ_r and a tangential stretch λ_t in the deformed state, expressed as

$$\lambda_\mathrm{r} = \lambda_0 \sqrt{(1 + u'(r))^2 + w'(r)^2} \tag{15}$$

$$\lambda_\mathrm{t} = \lambda_0 \left(1 + \frac{u(r)}{r}\right) \tag{16}$$

where $u'(r)$ and $w'(r)$ are the derivatives with respect to r. For a linear Saint Venant-Kirchhoff material, these stretches define the radial, tangential and initial stress, σ_r, σ_t and σ_0 respectively, via Young's modulus E and Poisson ratio ν.

$$\sigma_\mathrm{r} = \frac{E\,\lambda_0^2}{2\,(1-\nu^2)} \left(\left(\frac{\lambda_\mathrm{r}^2}{\lambda_0^2} - 1 \right) + \nu \left(\frac{\lambda_\mathrm{t}^2}{\lambda_0^2} - 1 \right) \right) + \sigma_0 \tag{17}$$

$$\sigma_\mathrm{t} = \frac{E\,\lambda_0^2}{2\,(1-\nu^2)} \left(\left(\frac{\lambda_\mathrm{t}^2}{\lambda_0^2} - 1 \right) + \nu \left(\frac{\lambda_\mathrm{r}^2}{\lambda_0^2} - 1 \right) \right) + \sigma_0 \tag{18}$$

$$\sigma_0 = \frac{E\,\left(\lambda_0^2 - 1\right)}{2\,(1-\nu)} \tag{19}$$

The stresses and shape of the elastomer membrane are given by a system of ordinary differential equations

$$\frac{\partial}{\partial r}\left(r\,\sigma_\mathrm{r}\left(1 + u'(r)\right)\right) - \sigma_\mathrm{t}\left(1 + \frac{u(r)}{r}\right) = \frac{p}{t_\mathrm{M}}\, w'(r)\;(u(r) + r) \tag{20}$$

$$-\frac{\partial}{\partial r}\left(r\,\sigma_\mathrm{r}\, w'(r)\right) = \frac{p}{t_\mathrm{M}}\left(u'(r) + 1\right)\;(u(r) + r) \tag{21}$$

with the boundary conditions

$$w'(0) = 0$$
$$w(a) = 0$$
$$u(0) = 0$$
$$u(a) = 0$$

For numerical calculations we use small values (e.g. 10^{-4}) for all dimensionless zero values, to avoid singularities. Solving these equations for different applied pressures gives the respective membrane shapes and enclosed volumes, resulting in the required pressure-volume characteristics, which can be used in further analysis. In particular, the paraxial focal length of the inflated membrane is calculated from the membrane shape[6].

Capacitance of a radially-symmetric electrostatic zipping actuator

To derive the electrostatic actuator energy W_A, an expression for the capacitance C is required. The actuator consists of a fixed bottom electrode with inner radius c and outer radius $c + l$ (radial length

[5] The *geometrical* nonlinearities are taken into account in this approach

[6] We calculate the focal length by ray tracing of parallel rays applying Snell's law. Rays within $10^{-2}a$ from the optical axis are considered and averaged.

l), and a top electrode with small slope (Supplementary Fig. S2). The initial opening angle between top and bottom electrode α_0 and is given by the actuator height h and length l.

$$h = l\,\tan\alpha_0 \tag{22}$$

When voltage is applied, the top electrode collapses from the outer edge, resulting in a zipped region with the radial length l_1 (and $l_\mathrm{L} = l - l_1$ for the unzipped region), and the opening angle changes to α. The capacitance of the actuator is the sum of the capacitance of the zipped region C_1 and of the unzipped region C_L.[7]

$$C = \underbrace{\frac{\varepsilon_\mathrm{F}\,S_1}{t_\mathrm{F}}}_{\text{zipped}\,C_1} + \underbrace{\int \frac{\varepsilon_\mathrm{F}\varepsilon_\mathrm{L}}{\varepsilon_\mathrm{L}t_\mathrm{F} + \varepsilon_\mathrm{F}t_\mathrm{L}(r)}\,\mathrm{d}S_\mathrm{L}}_{\text{unzipped}\,C_\mathrm{L}} \tag{23}$$

Here, S_1 is the area of the zipped region, S_L the area of the unzipped region, ε_F the permittivity of the plastic foil with thickness t_F, and ε_L the permittivity of the dielectric liquid with the radially changing thickness $t_\mathrm{L}(r)$. The first term describes the capacitance of the zipped region C_1 and can be expressed as a function of the displaced volume.

$$C_1(\Delta V) = \frac{\pi\varepsilon_\mathrm{F}}{t_\mathrm{F}}\,l_1(\Delta V)\,(2(c+l) - l_1(\Delta V)) \tag{24}$$

Assuming linear thickness dependence on radius $t_\mathrm{L}(r)$ (cone-shaped actuator), the integration in eq. (23) results in the capacitance of the unzipped region, which can be conveniently expressed via its outer radius $R = c + l - l_1$

$$C_\mathrm{L} = \frac{2\pi\varepsilon_\mathrm{L}l_\mathrm{L}^2}{h}\left(\left(\frac{R}{l_\mathrm{L}} + \frac{\varepsilon_\mathrm{L}\,t_\mathrm{F}}{\varepsilon_\mathrm{F}h}\right)\ln\left(1 + \frac{\varepsilon_\mathrm{F}\,h}{\varepsilon_\mathrm{L}t_\mathrm{F}}\right) - 1\right) \tag{25}$$

The capacitance ratio C_L/C_1 is large for small zipping regions with $l_1 << l_\mathrm{L}$. For a typical zipping regions with $l_1 \sim l_\mathrm{L}$ and $\varepsilon_\mathrm{L} \sim \varepsilon_\mathrm{F}$ this ratio is of the order of

$$\frac{C_\mathrm{L}}{C_1} \sim \frac{t_\mathrm{F}}{h}\ln\left(\frac{h}{t_\mathrm{F}}\right) \tag{26}$$

This ratio is small only asymptotically (for thin films with $t_\mathrm{F} << h$), and is often ~ 1 for typical numbers. However, for the *derivatives* with respect to ΔV (which enter the expression (6) for the pressure), $C'_\mathrm{L}(\Delta V)$ is usually much smaller than $C'_1(\Delta V)$ for all zipping lengths

$$\frac{C'_\mathrm{L}(\Delta V)}{C'_1(\Delta V)} < 0.1 \tag{27}$$

Neglecting the contribution of $C'_\mathrm{L}(\Delta V)$, and noting that the change in liquid volume $\Delta V_\mathrm{L} = -\Delta V$, we obtain:

$$\frac{dC(\Delta V)}{d\Delta V} \approx -\frac{dC_1(\Delta V_\mathrm{L})}{d\Delta V_\mathrm{L}} = \frac{6\,\varepsilon_\mathrm{F}}{h\,t_\mathrm{F}}\,\frac{R}{c+2R} \approx \frac{6\,\varepsilon_\mathrm{F}}{h\,t_\mathrm{F}}\,\frac{c+l}{3c+2l} \tag{28}$$

The last expression assumes moderate volume changes, so that R is close to its initial value $c+l$, which results in a constant $C'_1(\Delta V)$. This holds within few percent for our numbers. For large actuator sizes and small slope of the top electrode the contribution of C'_L increases and should be included in eq. (28). Within the same "moderate changes" approximation $R = c + l$ the derivative is modified as follows:

$$C' = C'_1 + C'_\mathrm{L} \approx \frac{6\,\varepsilon_\mathrm{F}\,(c+l)}{h\,t_\mathrm{F}\,(3c+2l)}\left(1 - \frac{(c+2l)}{(c+l)}\frac{t_\mathrm{F}\,\varepsilon_\mathrm{L}}{h\,\varepsilon_\mathrm{F}}\ln\left(\frac{h\,\varepsilon_\mathrm{F}}{t_\mathrm{F}\,\varepsilon_\mathrm{L}}\right)\right) \tag{29}$$

[7] SI units are used here, and for brevity ε_0 is included into all permittivities. The unzipped capacitance is calculated assuming two-layer capacitor with slowly varying liquid thickness t_L.

Displaced volume and the critical voltage

Substituting the result (28)[8] into the equality (6) together with the eq. (14) gives an analytic approximation for the equilibrium condition.

$$\frac{3\times 2^5}{\pi^3}\frac{\mu\, t_{\mathrm{M}}}{a}\left(\frac{V_0+\Delta V}{a^3}\right)^3 = \frac{6\,\varepsilon_{\mathrm{F}}}{h\, t_{\mathrm{F}}}\frac{c+l}{3c+2l}\frac{U^2}{2} \tag{30}$$

From here we obtain the dependence of the displaced volume on the applied voltage:

$$\Delta V = a^3\left(\frac{U}{U_{\mathrm{c}}}\right)^{2/3} - V_0 \tag{31}$$

Here, we defined the characteristic voltage U_{c}, which combines the majority of the system properties, but is separated from the basic scaling with respect to the aperture size a and the initial volume V_0.

$$U_{\mathrm{c}} = \left(\frac{2^5}{\pi^3}\frac{h\, t_{\mathrm{M}}\, t_{\mathrm{F}}}{a\,\varepsilon_{\mathrm{F}}}\frac{3c+2l}{c+l}\mu\right)^{1/2} \tag{32}$$

Equation (31) produces negative displaced volume ΔV for small voltages, which is forbidden by system's design. This means, that zipping does not start below some threshold (critical) voltage U_0:

$$U_0 = U_{\mathrm{c}}\left(\frac{V_0}{a^3}\right)^{3/2} \tag{33}$$

For small voltages $U < U_0$, the equilibrium state remains completely unzipped due to the initial filling V_0.[9]

Dependence of the focal length on voltage

Now we can relate the lens volume $V = \Delta V + V_0$ to its focal length f, and obtain its dependence on voltage. We use the thin lens expression $f = \rho/(n-1)$ for a plane-convex spherical cap of radius $\rho = (a^2 + Z^2)/2Z$, assuming small volume (lowest order in $Z/a << 1$). Here, n is the refractive index of the lens (paraffin oil). This is a good approximation for our system, which results in a simple relation:[10]

$$f(V) \approx \frac{\pi a^4}{4(n-1)}\frac{1}{V} \tag{34}$$

Together with the eq. (31), the focus-voltage relation for voltages $U > U_0$ becomes:

$$f(U) = \frac{\pi a}{4(n-1)}\left(\frac{U_{\mathrm{c}}}{U}\right)^{2/3} \tag{35}$$

For voltages below the threshold value, the focal distance remains unchanged and equal to its initial value $f(U < U_0) = f(V_0) = f_0$.

[8]The more accurate expression (29) with the corresponding changes was used in actual calculations, which modifies the result by a few percent.

[9]The situation is similar to Peano-HASEL actuators under a constant load [6]. For $U < U_0$ the free energy expression (5) has a minimum at $V_0 \neq 0$, $\Delta V = 0$, which has *positive derivative* with respect to ΔV.

[10]In a more detailed approach, one can develop parametric formulas, based on the full volume and pressure expressions (8), (10) and *thick* lens formulas for the focal length.

Relations between a deflected laser beam and the focal length

When a thin laser beam, parallel to the optical axis, enters the lens at the radial distance d_x from the axis, it is deflected by the angle β. This can be approximately treated as a single light ray. It hits the screen (positioned at a distance d_s behind the lens) at the distance y from the optical axis. These quantities are related to the focal length of the lens by:

$$\tan\beta = \frac{d_\mathrm{x}}{f} \tag{36}$$

$$y = d_\mathrm{x}\left(\frac{d_\mathrm{s}}{f} - 1\right) \tag{37}$$

When the focal length of the tunable lens changes by $f_2 - f_1 = \Delta f$, the tangent and the spot position on the screen change accordingly:

$$\Delta\tan\beta = \tan\beta_2 - \tan\beta_1 = -d_\mathrm{x}\frac{\Delta f}{f_1\, f_2} \tag{38}$$

$$\Delta y = y_2 - y_1 = d_\mathrm{s}\,\Delta\tan\beta = -d_\mathrm{x}\, d_\mathrm{s}\frac{\Delta f}{f_1\, f_2} \tag{39}$$

Therefore, the *relative* changes in $\tan\beta$ and spot position with respect to the reference state (1) are proportional to the relative change in focal length with respect to state (2).

$$\frac{\Delta\tan\beta}{\tan\beta_1} = -\frac{\Delta f}{f_2} \tag{40}$$

$$\frac{\Delta y}{y_1} = -\frac{\Delta f}{f_2}\frac{d_\mathrm{s}}{d_\mathrm{s} - f_1} \tag{41}$$

Both expressions do not depend on d_x, which simplifies the experimental measurements.

Cost estimation and scalability

A single small lens costs about 0.1431 € (calculation is given in Supplementary Table 3). The price is mostly determined by the PDMS elastomer, which makes 96 % of the total costs. The use of the elastomer could be readily reduced or replaced depending on the specific application or available fabrication facilities. Of course, the costs can be greatly reduced by the economies of scale as well. The lenses are scalable without changing the fabrication methods, as the lens body is molded and the polymer foils (laser) cut to dimensions. We note however that miniaturizing the lenses to the mm scale would require a change to fabrication schemes that are assisted by high precision machines.

Optimizing homogeneous zipping

The models derived above assume axial symmetric zipping of the polymers. However, variabilities of opening angles or initial distance of the polymer lead to inhomogeneous zipping in the experimental realization. We here use two approaches to better control the start and propagation of the zipping fronts.

Welding points: Regular separated welding points at the circumference of the actuator induce starting points for zipping, due to a locally reduced separation of the electrodes (Supplementary Fig. S4). As a rule of thumb, the distance between the welding points along the circumference should be (appreciably) smaller than twice the width of the zipping annulus. Likewise, in the presence of auxiliary concentric rings (see below), it should be smaller than twice the distance between these

rings. Increasing the number of welding points could lead to more homogeneous zipping, however only if the welding points are homogeneous as well. An infinite amount of points –hence a continuous circular welding– in practice still results in inhomogeneous zipping as certain regions of the welding will be (slightly) thinner and hence a (uncontrolled) starting point of a zipping front. Single points give better control over where the zipping starts.

Concentric rings: Concentrically patterned electrodes influence the zipping front propagation by inducing an anisotropy in radial direction. While the welding points represent a start of a zipping front, the concentric rings adjust the propagation speed of the zipping front. As we observed that the start of zipping fronts is much more important for homogeneous zipping than the propagation speed (at least for our lens dimensions), we disregarded the option of having concentric rings alone early in the design phase of the lenses.

Future work will investigate the influence of those approaches to optimize zipping of ZEAP actuators. Non circular electrodes, such as regular polygons, might also greatly enhance the zipping behaviour. Recent work suggests that edges act as starting points for zipping as well [7].

Towards ZEAP actuators with operating voltages <50 V

The characteristic voltage in eq. (32) relates to the operating voltages of ZEAP devices. Omitting weak influence of the actuators lateral size parameters c and l eq. (32) can be simplified to

$$U_\mathrm{c} \sim \left(\frac{\mu\, t_\mathrm{M}\, h\, t_\mathrm{F}}{a\, \varepsilon_\mathrm{F}}\right)^{1/2} \tag{42}$$

The remaining quantities can be divided into actuator parameters (h, t_F, ε_F) and lens membrane parameters (a, t_M, μ), but all parameters influence the characteristic voltage in similar extent. A reduction of t_F by a factor of 4 halves the operating voltages, as does a 4-times increase of ε_F. For the current design parameters $U_\mathrm{c} = 4.6\,\mathrm{kV}$, $3.1\,\mathrm{kV}$ for the small and large actuators respectively, which results in operating voltages $<500\,\mathrm{V}$. Keeping the macroscopic lateral geometry unchanged, the cumulative design parameters would need to be changed by a factor 100 to achieve a reduction of operating voltages to $<50\,\mathrm{V}$. This is possible by using softer membranes ($\mu^* = \mu/\sqrt{10}$) and foils with reduced thickness ($t_\mathrm{F}^* = t_\mathrm{F}/\sqrt{10}$) and higher permittivity ($\varepsilon_\mathrm{F}^* = 10\varepsilon_\mathrm{F}$).

Supplementary Tables

Author/ company	Lens material type	Actuation	Aperture (mm)	Minimum focal length (mm)	Maximum focal length (mm)	Driving voltages (V)
This work	liquid/ elastomer	electroactive zipping (EAZ)	6	22	550	<500
D. Wang *et al* [8]	liquid	electrowetting	15	15	165	<53
J. Wang *et al* [9]	liquid	electrowetting	6	19.2	265	<75
Jin *et al* [10]	liquid	inhomogeneous electric field	3	4.9	19.3	<52
Xu *et al* [11]	liquid	inhomogeneous electric field	3.5	-8.28	-4.4	<120
L. Wang *et al* [12]	elastomer	reshaping ring	1	950	∞	<5000
Bae *et al* [13]	elastomer	reshaping ring	1.5	3.8	22.3	–
Choi *et al* [14]	elastomer	reshaping ring	1.5	3	24.5	<400
Optotune 10-42-OF [15]	liquid	reshaping ring	10	-500	500	3.3
Wapler *et al* [16]	liquid/ solid	piezo electric	7.6	-142	167	–
Cheng *et al* [17]	liquid/ elastomer	electro hydraulic	6	24.5	690.3	<8000
Wirthl *et al* [18]	liquid/ elastomer	dielectric elastomer	20	32	67	<6500
Maffli *et al* [19]	liquid/ elastomer	dielectric elastomer	5	30.8	37.9	<3000
Carpi *et al* [20]	liquid/ elastomer	dielectric elastomer	7.6	16.72	22.73	<3500
Nam *et al* [21]	elastomer	dielectric elastomer	3	14.3	23.7	<4600

Table S1 | Overview of tunable lens systems and their performances.

Parameter	Notation	Unit	Small actuator	Large actuator
Pressure	p	kPa	0.2 - 200	
Aperture radius	a	mm	3	
Lens membrane thickness	t_{M}	mm	0.2	
Membrane prestretch	λ_0	–	1.0	
Inner actuator radius	c	mm	5	
Actuator length	l	mm	3	10
Outer radius	$c+l$	mm	8	15
Actuator height	h	mm	0.4	0.2
Zipping foil thickness	t_{F}	µm	6	
Zipped electrode length	l_1	mm	0 - 2.5	0 - 9.5
Young's modulus PDMS	$E=3\mu$	MPa	2.9	
Poisson's ratio PDMS	ν	–	0.5	
Refractive index paraffin oil	n	–	1.48	
Rel. permittivity paraffin oil	ε_{L}	–	3	
Rel. permittivity PET	ε_{F}	–	2.2	

Table S2 | Simulation parameters for the experimentally realized lenses. The values $n \neq \sqrt{\varepsilon_{\mathrm{L}}}$, as the permittivity is given for the quasi static case, while the refractive index refers to the optical frequencies.

Material	Quantity	Price (€/kg)	Costs (ct)
PDMS	0.8 g	171 €/kg	13.71 ct
Paraffin oil	62 µL	45.2 €/kg	0.28 ct
Copper	0.54 mg	5800 €/kg	0.32 ct
6 µm-thick PET foil	4 cm^2	–	< 0.01 ct
Total	–	–	14.3 ct

Table S3 | Material costs estimation. A single small lens costs about 0.1431 €. The price is mostly determined by the PDMS elastomer, which contributes to 96 % to the total costs. Prices are taken for typical research scale quantities (∼1 kg or 1 L packages).

Supplementary Figures

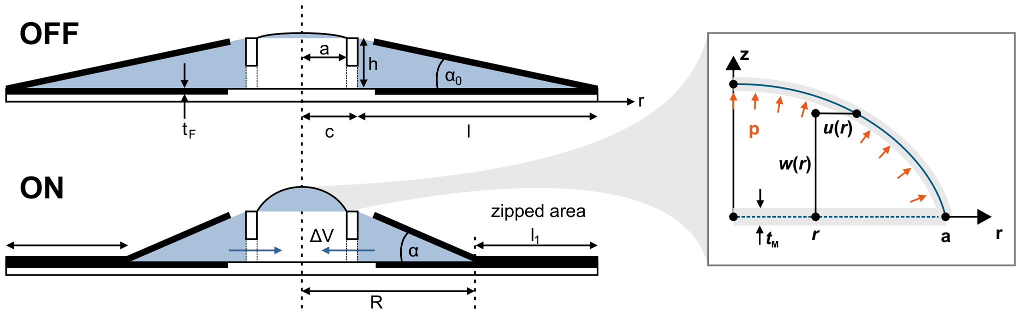

Figure S1 | Geometry parameters of a tunable lens. The volume ΔV displaced from the actuator into the lens, inflates the membrane. A point at position r is displaced by $w(r)$ in z-direction and $u(r)$ in lateral direction.

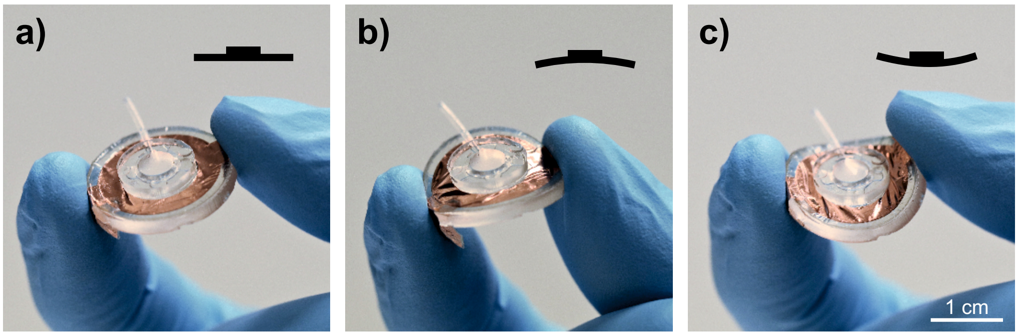

Figure S2 | Bending of soft tunable lenses. a) Flat tunable lens based on ZEAP actuators. The overall lens body is made of majorly soft materials and thin polymer foils. **b, c)** The lenses are bendable and compressible, which allows safe handling of the devices.

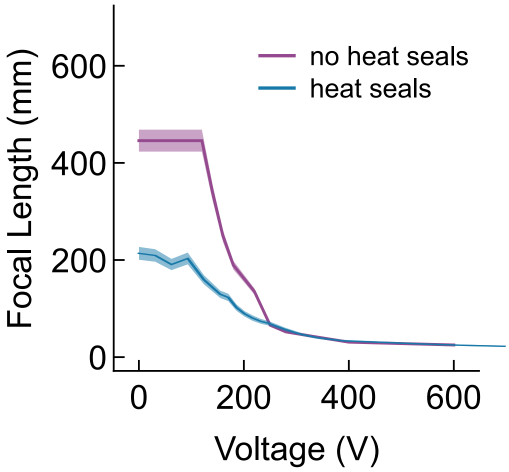

Figure S3 | Effect of heat seals. Comparison of lenses with large actuators featuring heat seals and no heat seals. Focal length changes require higher voltage in the initial regime ($<$300 V) when no heat seals are used. The focal length at higher voltages are unchanged. Note that the plateau, marking the threshold voltage, is at different initial focal length due to different initial fillings. The shape of the curves differs due to the effect of the heat seals (compare with Fig. 2g).

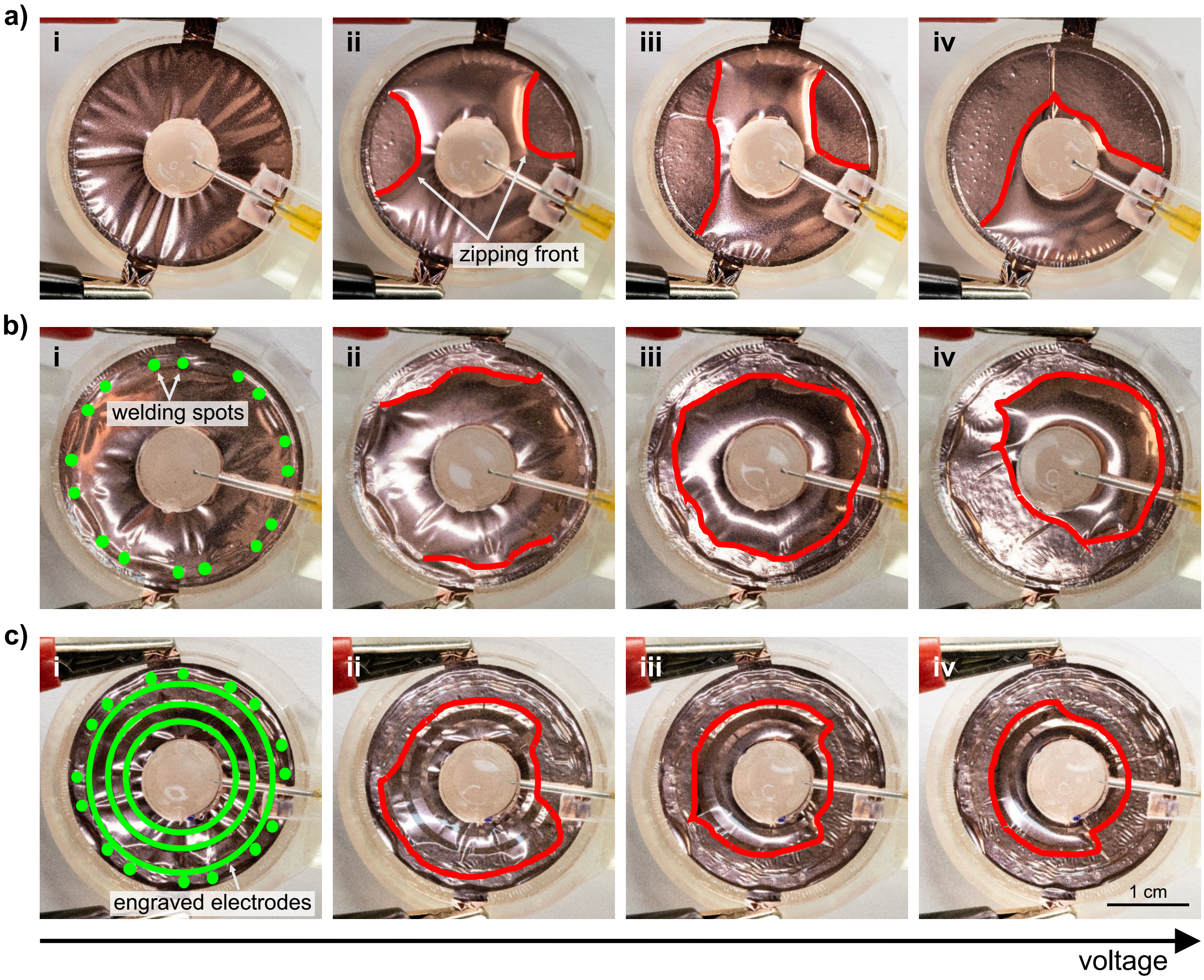

Figure S4 | Controlling the zipping behaviour. a), For large unstructured electrodes, zipping is not concentric and leads to partial inflation of the electrodes. The applied voltage increases from (i) to (iv). **b)**, Welding points facilitate the concentric zipping around the lens (iii). Still, zipping is dominant at specific sites (iv). **c)**, Additional concentric rings, engraved into the top electrode lead to more axially symmetric zipping. The rings are electrically connected via small copper traces left between them.

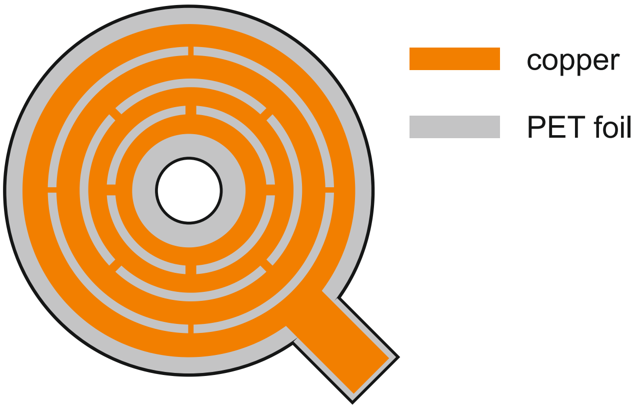

Figure S5 | Concentric rings pattern. A pattern of rings is laser-engraved into the copper electrodes to enhance concentric zipping.

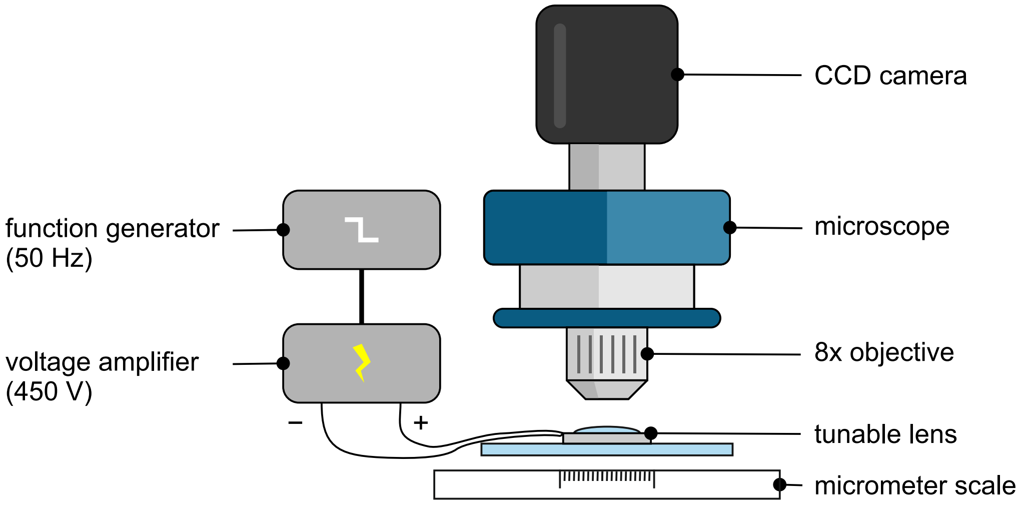

Figure S6 | Setup for focal length measurement. The tunable lens is positioned under a microscope with fixed distance to a micrometer-scale serving as image. The focal length of the tunable lens is calculated from the measured magnification. Abbreviations: CCD, charge coupled device.

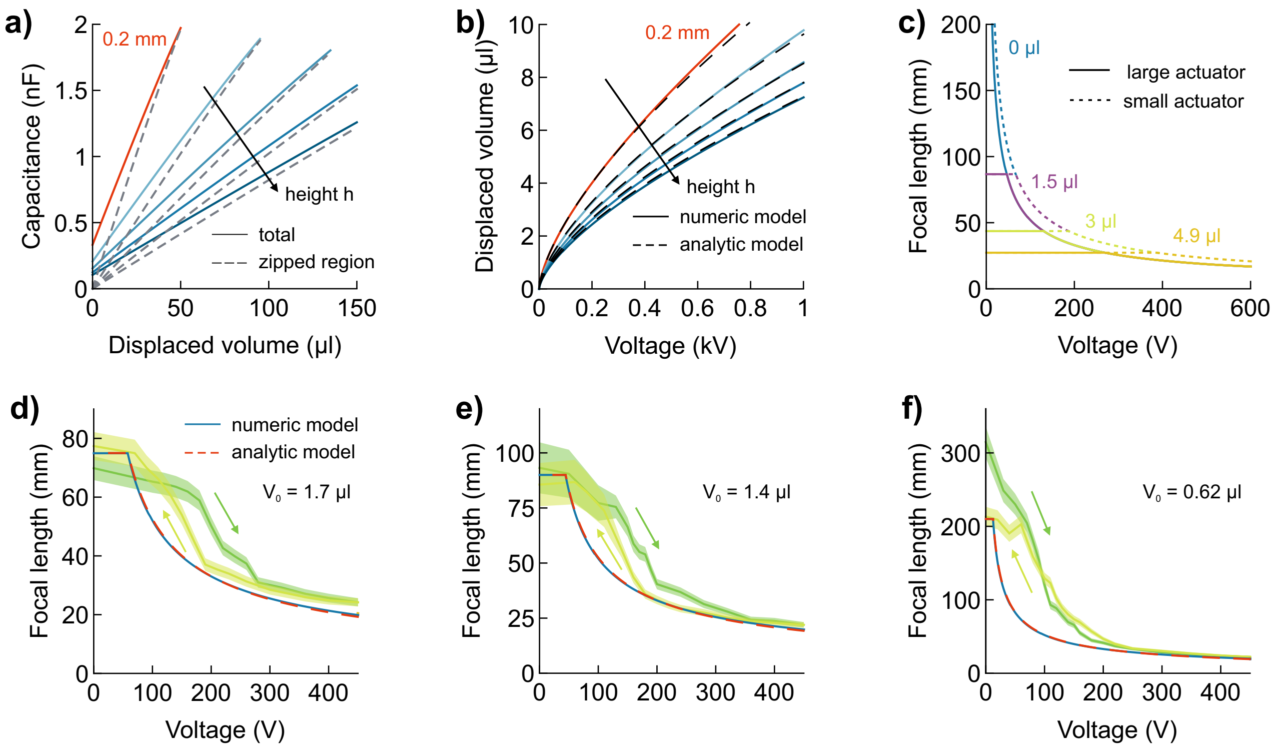

Figure S7 | Lenses with large actuators. a), The total capacitance of zipped and unzipped electrodes relates nearly linearly to displaced volume. Dashed lines represent the contribution of the zipped region. Characteristics are calculated for different actuator heights (0.2 (chosen height, red), 0.4, 0.6, 0.8, 1 mm). **b)**, The balance of mechanical and electrostatic forces results in the displaced-volume-voltage relations, with good agreement between the numerical (solid) and analytic (dashed) models **c)**, Combination of actuator and lens properties results in focus-voltage characteristics. The initial filling volume determines starting (maximal) focal length and the threshold voltage for focal length tuning. Lenses with a small actuator (dashed lines) require slightly higher operating voltages due to a larger initial opening angle compared to lenses with a large actuator (solid lines). **d) - f)**, Measured focal-length-voltage characteristics in comparison with the analytic (red dashed) and numerical (blue solid) models for lenses with different initial fillings. The experimental curves below and near the threshold voltages are influenced by effects that stem from non-uniform foil thickness due to welding spots or non-concentric zipping.

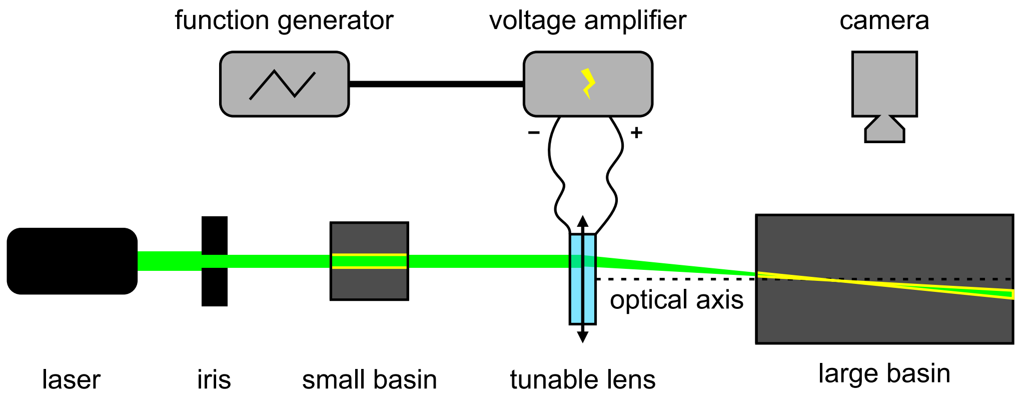

Figure S8 | Setup for dynamic response measurements. A laser beam is projected through a lens and two rhodamin-filled basins parallel to the optical axis at a fixed offset. The changing deflection of the laser beam is tracked with a camera at 50 frames per second.

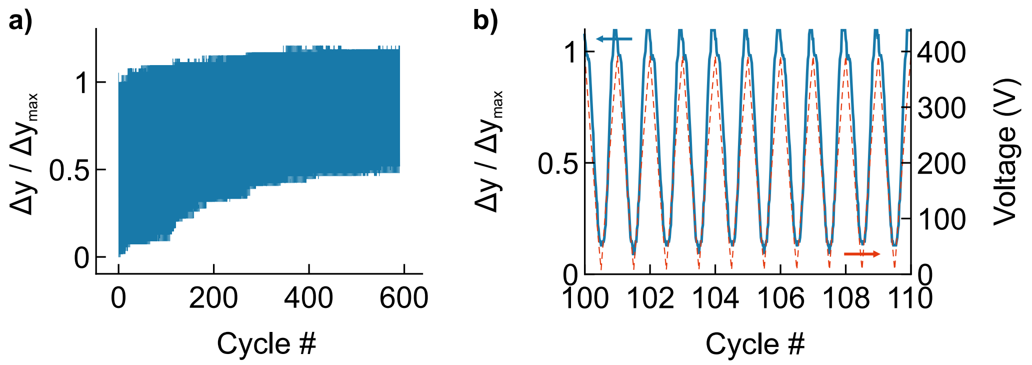

Figure S9 | Cyclic performance. a) Cyclic actuation of ZEAP tunable lenses shows small drifts and a slightly reduced performance after 600 continuous actuation cycles. **b)** The lens response follows the driving voltage, which is triangular in shape and changes polarity at every second cycle. No delamination of the PET foils can be observed over the course of the experiments.

References

[1] R. Baumgartner, A. Kogler, J. M. Stadlbauer, C. C. Foo, R. Kaltseis, M. Baumgartner, G. Mao, C. Keplinger, S. J. A. Koh, N. Arnold, et al. *Advanced Science* **2020**, *7*, 5 1903391.

[2] P. Rothemund, S. Kirkman, C. Keplinger. *Proceedings of the National Academy of Sciences* **2020**, *117*, 28 16207.

[3] Z. Suo. *Acta Mechanica Solida Sinica* **2010**, *23*, 6 549.

[4] C. Keplinger, M. Kaltenbrunner, N. Arnold, S. Bauer. *Proceedings of the National Academy of Sciences* **2010**, *107*, 10 4505.

[5] P. Pokornỳ, F. Šmejkal, P. Kulmon, P. Novák, J. Novák, A. Mikš, M. Horák, M. Jirásek. *Applied optics* **2017**, *56*, 34 9368.

[6] N. Kellaris, V. G. Venkata, P. Rothemund, C. Keplinger. *Extreme Mechanics Letters* **2019**, *29* 100449.

[7] E. Leroy, R. Hinchet, H. Shea. *Advanced Materials* **2020**, *32*, 36 2002564.

[8] D. Wang, C. Liu, Q.-H. Wang. *Optics express* **2019**, *27*, 7 10058.

[9] J.-H. Wang, X. Zhou, L. Luo, R.-Y. Yuan, Q.-H. Wang. *Optics Communications* **2019**, *445* 56.

[10] B. Jin, H. Ren, W.-K. Choi. *Optics Express* **2017**, *25*, 26 32411.

[11] M. Xu, D. Xu, H. Ren, I.-S. Yoo, Q.-H. Wang. *Journal of Optics* **2014**, *16*, 10 105601.

[12] L. Wang, T. Hayakawa, M. Ishikawa. *Optics Express* **2017**, *25*, 25 31708.

[13] J. W. Bae, E.-J. Shin, J. Jeong, D.-S. Choi, J. E. Lee, B. U. Nam, L. Lin, S.-Y. Kim. *Scientific reports* **2017**, *7*, 1 1.

[14] D.-S. Choi, J. Jeong, E.-J. Shin, S.-Y. Kim. *Optics express* **2017**, *25*, 17 20133.

[15] Optotune, **accessed: 13.07.2020**. URL `https://www.optotune.com/el-10-42-of-lens`.

[16] M. C. Wapler. *Optics Express* **2020**, *28*, 4 4973.

[17] X. Cheng, M. Yu, J. Ma, B. Li, Y. Zhang, P. Wang, Z. Jiao. *Smart Materials and Structures* **2020**, *29*, 4 045017.

[18] D. Wirthl, R. Pichler, M. Drack, G. Kettlguber, R. Moser, R. Gerstmayr, F. Hartmann, E. Bradt, R. Kaltseis, C. M. Siket, et al. *Science advances* **2017**, *3*, 6 e1700053.

[19] L. Maffli, S. Rosset, M. Ghilardi, F. Carpi, H. Shea. *Advanced functional materials* **2015**, *25*, 11 1656.

[20] F. Carpi, G. Frediani, S. Turco, D. De Rossi. *Advanced functional materials* **2011**, *21*, 21 4152.

[21] S. Nam, S. Yun, J. W. Yoon, S. Park, S. K. Park, S. Mun, B. Park, K.-U. Kyung. *Soft robotics* **2018**, *5*, 6 777.
